# AMMI and GGE biplot analysis for yield performance and stability assessment of selected Bambara groundnut (*Vigna subterranea* L. Verdc.) genotypes under the multi-environmental trials (METs)

**DOI:** 10.1038/s41598-021-01411-2

**Published:** 2021-11-23

**Authors:** Md Mahmudul Hasan Khan, Mohd Y. Rafii, Shairul Izan Ramlee, Mashitah Jusoh, Md Al Mamun

**Affiliations:** 1 Laboratory of Climate-Smart Food Crop Production, Institute of Tropical Agriculture and Food Security (ITAFoS), Universiti Putra Malaysia (UPM), 43400 Serdang, Selangor Malaysia; 2 Department of Crop Science, Faculty of Agriculture, Universiti Putra Malaysia (UPM), 43400 Serdang, Selangor Malaysia; 3 Bangladesh Agricultural Research Institute (BARI), Gazipur, 1701 Bangladesh; 4 Bangladesh Jute Research Institute (BJRI), Dhaka, Bangladesh

**Keywords:** Plant sciences, Plant breeding

## Abstract

The stability and high yielding of *Vigna subterranea* L. Verdc. genotype is an important factor for long-term development and food security. The effects of G × E interaction on yield stability in 30 Bambara groundnut genotypes in four different Malaysian environments were investigated in this research. The experiment used a randomized complete block design with three replications in each environment. Over multiple harvests, yield component traits such as the total number of pods per plant, fresh pods weight (g), hundred seeds weight (g), and yield per hectare were evaluated in the main and off-season in 2020 and 2021. Stability tests for multivariate stability parameters were performed based on analyses of variance. For all the traits, the pooled analysis of variance revealed highly significant (p < 0.01) variations between genotypes, locations, seasons, and genotypes by environment (G × E interaction). A two-dimensional GGE biplot was generated using the first two principal components (axis 1 and axis 2), which accounted for 94.97% and 3.11% difference in GEI for yield per hectare, respectively. Season and location were found to be the most significant causes of yield heterogeneity, accounting for 31.13% and 14.02% of overall G + E + G × E variation, respectively, according to the combined study of variance. The GGE biplot revealed that the three winning genotypes G1, G3, and G5 appear across environments whereas AMMI model exposed genotypes viz G18, G14, G7, G3, G1, and G5 as best performer. Based on ideal genotype ranking genotype G1 was the best performer, with a high mean yield and high stability in the tested environment. According to the AEC line, genotypes G1 and G3 were extremely stable, while genotypes G2 and G4 were low stable, with a high average yielding per hectare. A GGE and AMMI biplot graphically showed the interrelationships between the tested environment and genotypes, classified genotypes into three categories as well as simplifying visual evaluations, according to this investigation. According to our results, breeding could improve yield production, and the genotypes discovered could be recommended for commercial cultivation.

## Introduction

Bambara groundnut [*Vigna subterranea* L. (Verdc.)] (2n = 2x = 22) is an under-research legume crop belong to the family Fabaceae and subfamily Faboideae of genus *Vigna*^1^. It originated from West Africa and is still a traditional food crop in the African continent^2^, besides Africa, it is successfully farming in the Asia and South-Asian region like Malaysia, Thailand, Indonesia, Phillippines, Srilanka, India, and Brazil also^3^. It is commonly known as “Okpa” (Igbo; eastern Nigeria), “Epa-Kuta” (Yoruba; western Nigeria), “Kacang poi” (Malaysia), and very recently treated as a new Millennium crop^2,4^. Bambara groundnut is a legume, which is tolerant to drought and adapted to poor soils where other crops are not adopted^2,5^. As a source of ‘complete food’ dried Bambara seeds contain significant portion of essential macro-nutrients (CHO: 49–63.5%; protein: 15–25%; fat: 4.5–7.4%; fibre: 5.2–6.4%; ash: 3.2–4.4% and minerals: 2%) and micronutrients (calcium: 95.5–99.0 mg; iron: 5.1–9.0 mg; potassium: 11.45–14.36 mg and sodium: 2.9–10.6 mg) per 100 g seed weight^6^. It is the third most promising legume in Africa, behind groundnut and cowpea^4^, and is mostly used for human consumption^7^ as well as livestock fodder^8^. Through nitrogen fixation, the crop contributes to improved soil fertility^9^. Surplus Bambara groundnuts are often traded in local markets, thus generating revenue for resource-limit farmers^10^. The west African region Mali produced the highest amount (1,45,240 Mt) of Bambara ground whereas on average 1.10 t ha^−1^ is the maximum production by Burkina Faso^11^. In subtropical climate, average production was accounted for 1.18 t ha^−1^^4^ and 0.38 to 1.6 t ha^−1^^3^ in Malaysia, 0.7 to 2.0 t ha^−1^ by Redjeki^12^ in Indonesia. At optimal farming circumstances, it has the ability to produce up to 4.0 t ha^−1^ of dry grain^13^. However, the remerkable quantity of production is tough due to the crop cultivated for subsistence purposes and often as mixed and intercrop with other cereals. The major drawbacks of this legume expansion can be enlightened by some factors such as lack of modern production scheme and commercial high yielding cultivars hence growers still used to cultivates traditional landraces^14^. Despite its great nutritional content and ability to grow and thrive in poor soils, it is considered one of the world's most neglected crops by the World Scientific Forum^15^. In Malaysia, the lack of high-yielding Bambara groundnut cultivar is one of the major burdens to its production. Plant breeders have employed diverse methods in developing progressive Bambara groundnut cultivars using traditional and advanced molecular methods^15^.

Breeders often use yield and its contributed performance as well as a phenotypic expression for sorting and selection of crop cultivars under mega environment test. The vital efforts in crop enhancement methods are focused on risk minimization, yield stability, cost savings, and maximisation of revenues^19^. Pathogenic infections, humidity, soil texture and fertility, precipitation, and temperature may all play a role in the yield flactuation caused by genotypes' responses to changing environments^16^. This yield instability or fluctuation is named genotype by environment interaction (GEI) reported in different crops^17,18^. The opportunity of increasing Bambara grain yield is authoritative to improve yield stability. However, for newly generating advance lines, a prerequisite to undertaking a multi-environment yield trail to determine the superior and stable Bambara groundnut genotypes adapted in the growing region of multi environments. To determine the presence of GEI in a multi-environmental yield trial Analysis of variance (ANOVA) is executed. The ANOVA procedures highlighted differentiation in fixed and random effects such as genotype, replication, location, and environment. Nevertheless, the major bottlenecks of ANOVA are the failure to distinguish genotype variances in a non-additive manner as G × E interaction^19^. A group of stability statistical measures was settled to analyze genotype stability that divulges several G × E interaction features, resultant in detecting stable genotypes across environments. To get a better understanding of genotypic stability patterns, two separate techniques such as univariate and multivariate of stability statistical analysis are used^19^. Multivariate methods like as cluster analysis, pattern analysis, and principal component analysis or biplots are effective tools for uncovering patterns of GE interactions^20^. The pattern analysis (combination of classification and ordination technique) will be efficient in uncovering and elucidating the GEI structure of intrinsic data under examination^21^. Biplots are widely used to graphically show the interrelationships between genotypes (G), environments (E), and GEIs, as well as to demonstrate interaction patterns and identify the comparably stable genotypes across environments^19^. Principal component analysis (PCA), also known as biplots, is a popular graphical representation of interaction outlines used to reveal inter-relationships between genotypes, environments, and GEI in order to identify genotypes that are well suited to specific environments or genotypes that are stable across environments. The two-way measures such as GEI data to singular value decomposition (SVD) and its graphical display are subjected to generate the biplots. There are two widely used biplot models are AMMI biplot = the additive main effects and multiplicative interaction and GGE biplot = genotype + genotype × environment. The analysis of variance of the genotype and environment main effects with the PCA of the GEI generates the AMMI model while the AMMI2 or GEI biplot is performed based on the SVD of a double centered G × E table^22^. Kaya et al.^23^ and Admassu et al.^24^ validated the effectiveness of the AMMI technique to identify the discriminating genotypes that had stable performance across diverse environmental conditions. The GGE biplots are based on the singular value decomposition (SVD) of environment-centered provided by Yan et al.^25^ and graphically represent both genotype and genotype-by-environment based on primary sources of variation associated to genotype assessment. GGE biplots-based multi-environment trail (MET), genotype evaluation environmental valuation has been successfully executed for varietal stability analysis experimented by several researchers such as Mohammadi et al*.*^26^, Kumar et al*.*^27^, and Mogale^11^. Contrarily, the GGE biplot approach for decomposing genotype plus genotype-by-environment (G + G × E) is censured by Gauch et al.^28^ however, the observation of G + G × E is more effective in biplot graph compared to AMMI analysis. GGE biplot analysis has been used by several researchers to classify the mega environment, assess genotype rankings, and decide the discriminative and representative among the tested environments^19^. During yield trials, an additive main effect and multiplicative interaction (AMMI) model is widely used to analyze G × E interaction^19^. It is important to understand how GE interacts with cultivars in order to determine adaptation and stability^29^. AMMI is capable of detecting GEI in a multi-dimensional environment and displaying it using a biplot. The GGE biplot will aid researchers in better understanding complicated GE interactions in multi-environment breeding line trials and agronomic investigations^29^. The GGE biplot was used to determine the performance of crop cultivars in a variety of stress conditions, ideal cultivars, mega-environment, and core testing sites^30^. Direct presentation of genotype effects is not feasible in AMMI2 biplots since this approach only decomposes G × E interaction effects in the PCA. GGE biplots analysis, on the other hand, is regarded as a useful statistical technique for producing phenotypically stable and superior cultivars, identifying stable genotypes across several environments, and achieving crop yield stability across multiple locations. As a consequence, the current study aims to identify superior genotypes with stable yield performance over a wide range of environments by evaluating the efficacy of various stability analysis methodologies. Another intention of this study was to examine how GEI influenced the yield and yield components of *Vigna subterranea* L. (Verdc.) genotypes as well as to identify the high yielding stable genotypes for future breeding schemes in tropical and subtropical environments.

## Materials and methods

### Plant materials

The research work was conducted under the Institute of Tropical Agriculture and Food Security (ITAFoS), University Putra Malaysia (UPM), Malaysia. In this work, selected 30 V*. subterranea* accessions from the GenBank of ITAFoS, UPM were utilised. Initially, fifteen collected accessions were formal identified and investigated by Md Mahmudul Hasan Khan^3,4^ under the direct supervision of Prof. Dr. Mohd. Rafii Yusop, Director, ITAFoS, UPM, Malaysia, in accordance with appropriate national and international policies. During the assessment, potentiality of high yield was considered to choose the accessions from each selfed generation of S_0_ to S_5_. However, we chose 150 individual plants from the fifteen assessed landraces of generation S_0_ based on the greatest number of pods and yield per plant and subjected them to subsequent selfing and selection as S_1_ selfed generation. These seeds from S_1_ selfed generation were cultivated for selfing and promoting the next generation as S_2_, and the top 44 performing lines were chosen. Similarly, following two rounds of selfing and selection (viz. S_2_ and S_3_), we sowed the seeds of the S_3_ and S_4_ generations together for a comparative and inbreeding depression assessment. Moreover, molecular characterization (https://doi.org/10.1038/s41598-021-93867-5) also executed by Khan et al.^15^ using the 44 accessions of S_4_ selfed generation. However, the seeds of all potential lines are stored in GenBank, ITAFoS, UPM. Finally, we picked the 30 best-performing lines of *V. subterranea* from the 44 accessions of the fourth selfed (S_4_) generation based on high yield and phenotypic uniformity and considered them to be the S_5_ selfed generation. We collected the plant seeds or specimens with the proper permission of the institution's authority by following the national and international strategies as well as deposited them in GenBank, ITAFoS, UPM. We also took appropriate permission from farm or field owner during specimens’ collection and experimentation in Malaysia. The name and ID of each accession were listed in Table 1.

**Table 1:** List of selected genotypes used in this study.

Genotype	ID	Genotype	ID	Genotype	ID
MaikP12-18	S5G1	GiiwP12-18	S5G11	GiiwP9-18	S5G21
MaikP3-18	S5G2	ExSokP4-18	S5G12	GiiwP11-18	S5G22
MaikP6-18	S5G3	KarP10-18	S5G13	KarP8-18	S5G23
BdilaP5-18	S5G4	MaikP11-18	S5G14	DunP6-18	S5G24
JataP1-18	S5G5	MaibP8-18	S5G15	GiiwP1-18	S5G25
DunP9-18	S5G6	MaibP6-18	S5G16	KataP5-18	S5G26
CancP3-18	S5G7	KataP8-18	S5G17	KarP9-18	S5G27
RokP1-18	S5G8	DunP2-18	S5G18	DunP8-18	S5G28
ExSokP5-18	S5G9	CancP2-18	S5G19	RokP9-18	S5G29
ExSokP3-18	S5G10	BdilaP8-18	S5G20	JataP3-18	S5G30

*Maik* Maikai, *Bdila* Bidillali, *Jata* Jatau, *Dun* Duna, *Canc* Cancaraki, *Rok* Roko, *ExSok* Exsokoto, *Giiw* Giiwa, *Kar* Karu, *Maib* Maibergo, *Kata* Katawa.

### Environments and inter-cultural practices

The field trials were directed recurrently across two locations in two cropping seasons (2020 and 2021) in Malaysia. The environments (combination of seasons and location) spanned a considerable degree of conditions varied in temperature (warm vs. moderate climate), rainfall (heavy rain vs. additional irrigation), soil structure, soil pH, and management practices (research's vs. farmer’s field). Details of the environmental conditions were presented in Table 2. The soil properties of the experimental site are listed in Table 3.

**Table 2:** Environmental description of the experimental site.

Code	Season	Latitude	Longitude	Altitude	Av Temp	Av Hum (%)	Rainfall (mm)	Year
FTM	Main	2.990935	101.7138	61.0 m	23.14–29.88 °C	83.2	188.6	2020
FTO	Off	2.990935	101.7138	61.0 m	24.22–30.72 °C	82.6	198.4	2021
FFM	Main	2.983092	101.7152	54.0 m	23.14–29.88 °C	83.2	188.6	2020
FFO	Off	2.983092	101.7152	54.0 m	24.22–30.72 °C	82.6	198.4	2021

*FTM* Field 10 main season, *FTO* Field 10 off season, *FFM* Field 15 main season, *FFO* Field 15 off season, *Main season* May–September, *Off season* November–March, *Av. Temp.* Average temperature, *Av. Hum.* Average humidity. Sources: https://en.climate-data.org/asia/malaysia/selangor/mardi-serdang-971613/#climate-table.

**Table 3:** Characterization of soil properties of the experimental region.

Site	Clay (%)	Sand (%)	Silt (%)	pH	References
Field 15	33.74	40	26.82	6.6–7.5	Hashemi et al.^31^, Khan et al.^4^
Field 10	62	31.83	6.4	4.23–4.6	Fahmi et al.^32^

The experiment was set up in a randomized complete block design (RCBD) with three replications in each environment. The experimental plot was divided into two rows of 1.6 m × 0.80 m each. According to Khan et al.^3^, the distance between plants was 30 cm, row to row was 50 cm, plot to plot was 1.5 m, and the distance between replication was 2.0 m. Recommended intercultural activities such as field planning, land clearing, weeding, irrigation, and fertilizer were used during the growing season. The prescribed fertilizer rates (100% N = 45 kg N/ha, 100% P = 54 kg P_2_O_5_/ha, 100% K = 45 kg K_2_O/ha) and all portions of Phosphorus and Potassium were applied during final land preparation, though, 70% N was added at five weeks after planting^3^. The field was mechanically plowed in the study places, following the usual cultural traditions of the local farmers. Where the need arose, pest and disease control was carried out. Hand weeding was done as needed, and systemic herbicide was used to control broad leaf weeds prior to land preparation and around the experimental plot.

### Data collection

For this study, we considered four quantitative traits (direct and positively contributing traits with yield) such as total number of pods per plant (TNP), fresh pod weight per plant (g) (FPW), hundred seed weight (g) (HSW), and yield (kg/ha). However, data were recorded according to Bambara groundnut classification and descriptors by IPGRI, IITA, and BAMNET^33^. Data was collected from 5 randomly selected plants from each plot in each replication at various growth stages in the field and the plant physiology lab after harvest.

### Statistical analysis

The quantitative traits were subjected to analysis of variance (ANOVA) to estimate the existence variations among the genotypes, locations, seasons, genotype by location, genotype by season, and genotype by location by season (genotype by environment) using SAS version 9.4. The genotypes were treated as fixed variables, while the environments were considered random variables. An additional statistical analysis was carried out if there is a significant interaction between the environment and the genotype to determine the stability level among the 30 genotypes across environments. The G × E SAS code developed by Dia and Wehner^34^ was used for stability analysis which is freely available at http://cuke.hort.ncsu.edu/cucurbit/wehner/software.html. The G × E SAS output consists of ready to use input file in R-package for multivariate analysis. To explain the G × E interaction, the multivariate stability analysis was performed graphically based on GGE biplot and AMMI using R studio (a simplified version of R statistical software) developed by the R Core Team^35^. The GUI package of R studio was used for GGE biplots while the Agricolae package was used for AMMI^36^, involving two concepts, the biplot concepts^37,38^ and the GGE concept^25^. The GGE biplots and AMMI are graphical images to exemplify G × E interaction and genotype ranking based on mean and stability. The graph generated is based on multi environment evaluation (which-won-where pattern), Genotype evaluation (mean versus stability), and tested environment raking (discriminative versus representative). The ranking of genotypes was allocated in increasing order of each stability parameter. The biplots were based on singular-value partitioning = 2, transformed (transform = 0), environment-centered (centering = 2), and standard deviation-standardized (scaling = 0).

## Results and discussion

### Combined variance analysis for yield and its related traits

To describe the main effect and quantify the interactions among and within the source of variations combined analysis of variance was performed. The pooled analysis of variance was displayed in Table 4. The mean square of locations, seasons, genotypes, and genotypes by locations by seasons (G × L × S; i.e., G × E interaction) showed significant differences (p ≤ 0.01, p ≤ 0.05) for TNP, FPW, HSW and yield per hectare. The genotypes by seasons (G × S) and genotypes by locations (G × L) had no significant variation for the TNP and HSW while the trait FPW and yield had a significant difference for genotypes by locations (G × L). Highly significant differences in locations, seasons, and genotypes may be attributed to changes in environment conditions and genetic makeup that differ from one environment to the next. The partitioning of the percentage of G × E interaction (% of GE) is computed from the total sum of the square shown in Table 4 which elucidates the percentages of variation for all traits. Except for the total number of pods (TNP), other traits showed a considerable extent of variation due to location that spanned from 0.15 to 14.02%. A greater difference among locations for genotype means resulting in most of the variation presence in genotype performance. Oppositely lower variation was found in genotype by location which varied from 0.61 to 7.20%. The trait total number of pods (22.9%), fresh pod weight (21.67%), and yield (22.4%) had near similar contribution towards genotype effect while hundred seed weight (7.34%) had small variation due to genotype effect. The genotype by environment interaction (GEI) i.e., G × S × L effect accounted for 9%, 8.52%, 2.52%, 1.85% of the total sum square for the traits total number of pods, fresh pod weight, hundred seed weight, and yield per hectare, respectively. The location by season effect had 68.66% variation for hundred seed weight, season effect contributed 31.13% and 42.64% variation for the trait yield per hectare and fresh pod weight respectively. Seasons contributed 31.13% variation for yield per hectare indicated that the assessed seasons in this study were different, which is highly attributed by the trait yield per hectare although G × E interaction significantly contributed 1.85%. The percent of the sum of the squares for the location by season (L × S) impact in yield per hectare is larger than the magnitude of the location effect, suggesting that there was a considerable degree of variation across the location due to two growing seasons. Similar findings were reported by Oladosu et al*.*^19^ stated that 29.07% variation contributed by location effect for grain yield. In this current research, a significant level divergence among G × E interaction and genotype effect indicated the certainty of the presence of diverse multi-environments with different genotype as well as high yield potential^39^. Nevertheless, the variance component analysis is not enough to clarify the details of the genotype by environment interaction. Henceforth, additional statistical techniques such as multivariate analysis can be more fruitful in unfolding and understanding the GEI^19^. The genotype by environment interaction effect primarily highlights the fact that genotypes responded inversely to various locations, emphasizing the need of genotypes assessment in diverse environments. Likewise, GEI reveals the challenges that plant breeders have, when identifying a superior genotype for commercial farming before releasing it as a variety^19^. The environment's partition of variance component revealed that predictable (locations) and unpredictable (seasons) aspects were important sources of variation^19^. When GEI is subjected to the effect of predictable components, plant breeders can either choose genotypes for a specific environment or extensively adjusted genotypes across several environments^40^.

**Table 4:** Estimation of significant level for yield and yield contributed traits of 30 V*. subterranea* accessions revealed by ANOVA.

SOV	df	TNP		FPW		HSW		Yld kg/ha	
		MS	TSS%	MS	TSS%	MS	TSS%	MS	TSS%
Reps (location)	4	416.98**	5.97	17,070.8**	2.67	1805.23**	0.93	160,078.81**	2.24
Locations (L)	1	41.78ns	0.15	154,052**	6.02	34,081.11**	4.44	4,005,612.29**	14.02
Seasons (S)	1	1117.73**	4.00	1,090,765.81**	42.64	65,640.72**	8.54	8,896,131.79**	31.13
L × S	1	1095.72**	3.92	11,182.73**	0.44	527,565.36**	68.66	5,791,601.23**	20.27
Genotypes (G)	29	220.44**	22.90	19,117.35**	21.67	1945.19**	7.34	220,685.25**	22.40
G × S	29	59.49ns	6.18	7289.823	8.26	189.89ns	0.72	8466.66ns	0.86
G × L	29	69.31ns	7.20	5905.88**	6.70	143.52ns	0.54	6005.29*	0.61
G × S × L	29	86.64*	9.00	7510.57**	8.52	668.26**	2.52	18,272.78**	1.85
Error	236	48.10	40.66	333.34	3.08	204.92	6.29	8017.25	6.62

*SOV* source of variation, *df* degree of freedom, *TNP* total number of pod, *FPW* fresh pod weight, *HSW* hundred seed weight (g), *Yld* Yield (kg/ha).

*Significant at p ≤ 0.05; **highly significant at p ≤ 0.01; ns = non-significant p > 0.05.

### Biplot pattern for elucidation of multivariate analysis

Globally, crop farming in absence of G × E interaction is performed equally, thereby having a common result irrespective of the environment^19^. In a statement reported by Yan et al*.*^25^, the main effect of genotype (G) plus G × E interactions is the principal source of variation in the assessment of the genotypes under multi-environment trials (MET). Three major components can be elucidated using the biplot such as (a) ‘which-won-where’ pattern or MET, proposed by Yan et al*.*^25^ is an effective approach to visualize the pattern of GEI based on the correlation between G and E; (b) stability vs mean performance over the environment for genotype evaluation; (c) representativeness and discriminating ability for test environment assessment. The prefix ‘Bi’ in the word biplot denotes the dual (genotypes and environment) exposing on the same graph. Biplot is a 2D visualization matrix that has two axes, first data was centered afterward sectionalizing the singular value (SV) into GE scores for individual principal components viz. PC1 and PC2 followed by intrigue the PC1 scores contrary to the PC2 scores to create a biplot^19^. The greater PC1 value indicates greater yielding ability whereas the lower PC2 value signifies stability. A biplot is made up of an asymmetrical polygon with stripes or lines running vertically from the biplot's centre to the polygon at a right angle. All the genotypes that are apart from the biplot center are linked with the polygon thus covering all genotypes in the polygon marker. The vertical stripe that runs perpendicular to the polygon from the centre of the biplot represents an expected environment in which the two genotypes on opposite sides of the polygon are expected to behave similarly^19^. Furthermore, it divides the biplot into different parts, each with its own enticing or winning genotypes^19^. The captivating genotype is always positioned at the vertex of the polygon where both sides of the polygon meet that vertical stripe, generate a borderline of that segment or section^41^.

#### GGE biplot (‘which-won-where’ pattern)

**Figure 1:**
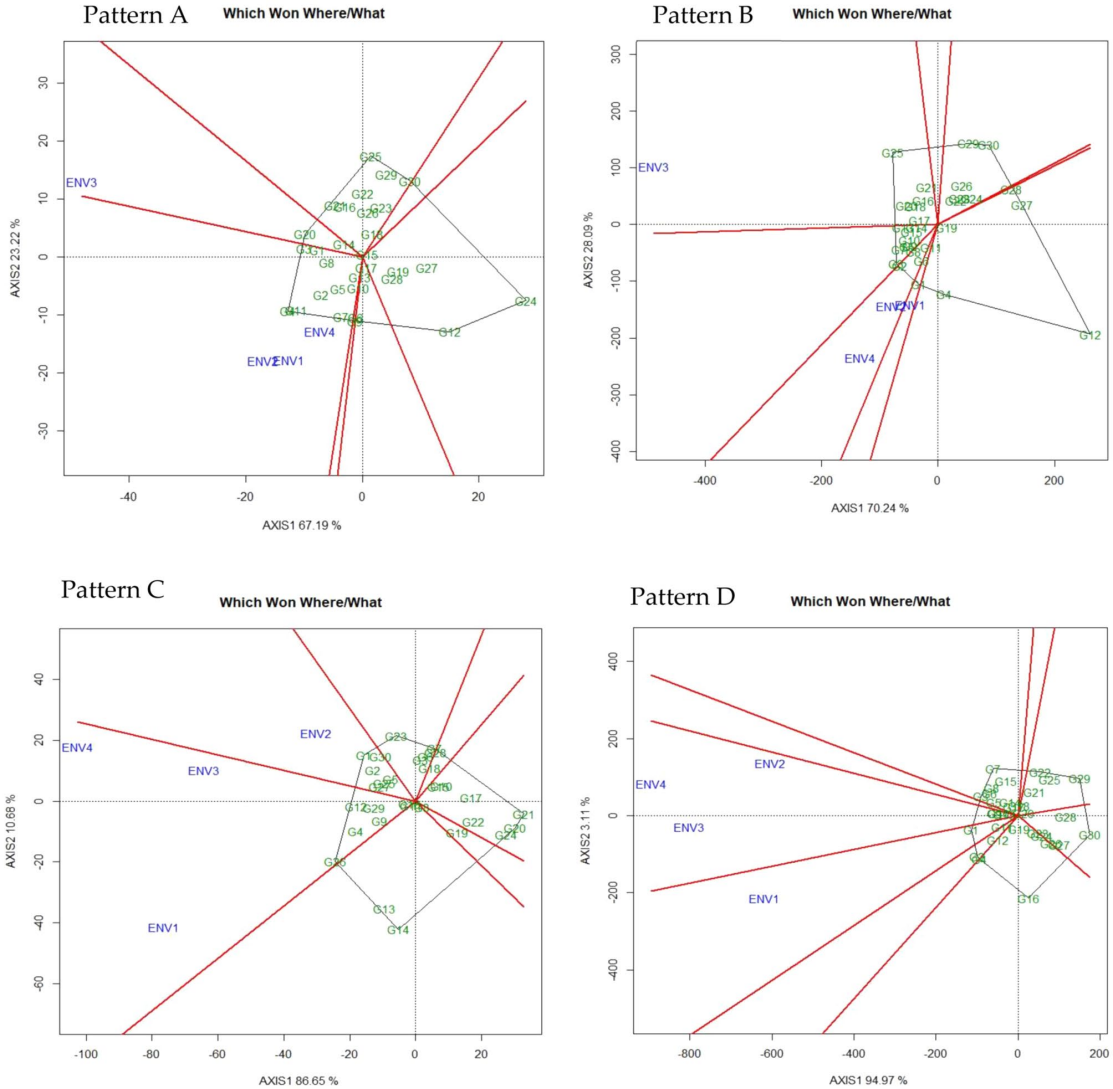
“Which-won-where” pattern of GGE biplot polygon view displaying the genotype main effect plus G × E interaction effect of 30 V*. subterranea* genotypes in two seasons two location for total number of pods (pattern A), fresh pod weight (Pattern B), hundred seed weight (Pattern C), yield per hectare (Pattern D). The biplots were based on Centering = 0, SVP = 2, Scaling = 0. The key to the genotype labels and the environmental description is given in Tables 1 and 2, respectively.

Figure 1 illustrated the polygon view of the GGE biplot pattern for total number of pods (pattern A), fresh pod weight (Pattern B), hundred seed weight (Pattern C), yield per hectare (Pattern D). The G + G × E variation was recorded as 90.41%, 98.33%, 97.33%, and 98.08% for TNP, FPW, HSW and Yield, respectively (Fig. 1, Pattern A, B, C, D). The environmental indicators positioned into 2, 3, 2, and 2 segments or sections of biplot for TNP, FPW, HSW, and Yield, respectively with different genotype winning in each segment. This result confirming the presence of distinct interaction between genotype and environment for all the traits evaluated. Based on 30 genotypes and 4 environments the generated GGE biplot was divided into 7, 8, 7, and 9 clockwise fan-shaped sections for TNP, FPW, HSW, and Yield, respectively. The genotype G20 produced a maximum number of pods and highly stable in ENV3 while genotypes G3, G4, G7, and G11 perform best in ENV1, ENV2, and ENV3. The genotype G25 in ENV3, genotype G2 in ENV2 and ENV4, genotype G1 in ENV1 were recorded as highly stable and produce more fresh pods. For hundred seed weight genotype G26 and G12 in ENV 1, ENV 3, ENV 4 whereas genotype G1 in ENV 2 was found as highly stable and best performing line. However, the genotype G1 was recorded as high yielding and stable genotype for environment one (ENV1). The findings of our study are the agreement with the report stated by Hashim et al*.*^48^ considered two seasons two location and Oladosu et al*.*^19^ considered two seasons five location. The positioning of all environmental indicators into one section of biplot directed that a unique genotype performs best under all tested environments. Oppositely, different genotypes gained different environments if the environmental indicators were positioned into a different segment of biplot. Besides, the genotypes placed at the polygon vertex in a section of biplot where there is no environmental indicator are treated as poorly perform genotypes under all tested environments^19^. Consequently, exposing the ‘which-won-where’ pattern of the GEI data matrix is a crucial feature of the GGE biplot that was extracted by the innermost assets or product of the biplot^39^. The genotype that attached with a vertex of the polygon in a sector where environment markers drop in suggested, such genotype provided greater yield and perform best in that environment. On the contrary, a genotype that is linked with polygon vertex where no environment indicator drops in the sector indicated that such genotype is poorly performed across the environment. The genotypes placed within the polygon are less respective to the environment than the corner genotypes. However, if multi-environments acknowledge by different winning genotypes recommends the presence of GEI in 4 environments for TNP, FPW, HSW, and Yield, this trend is validated by Gauch and Zobel^42^.

#### GGE biplot pattern of ‘mean vs. stability’ analysis and ideal genotype assessment

**Figure 2:**
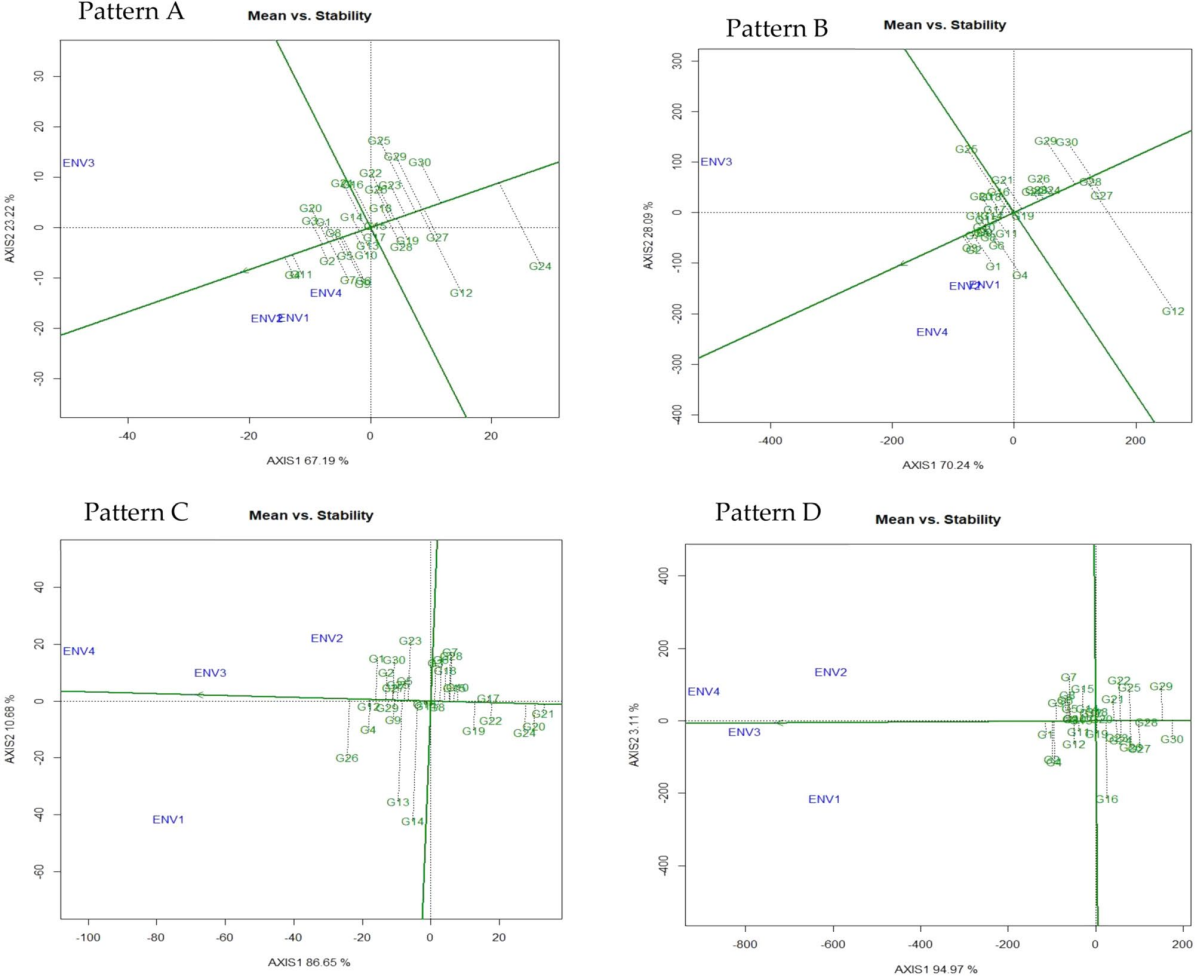
‘Mean vs. stability’ pattern of GGE biplot illustrating interaction effect of 30 V*. subterranea* genotypes under two season two location for total number of pods (Pattern A), fresh pod weight (Pattern B), hundred seed weight (Pattern C), yield per hectare (Pattern D). The biplots were created based on Centering = 0, SVP = 2, Scaling = 0.

**Figure 3:**
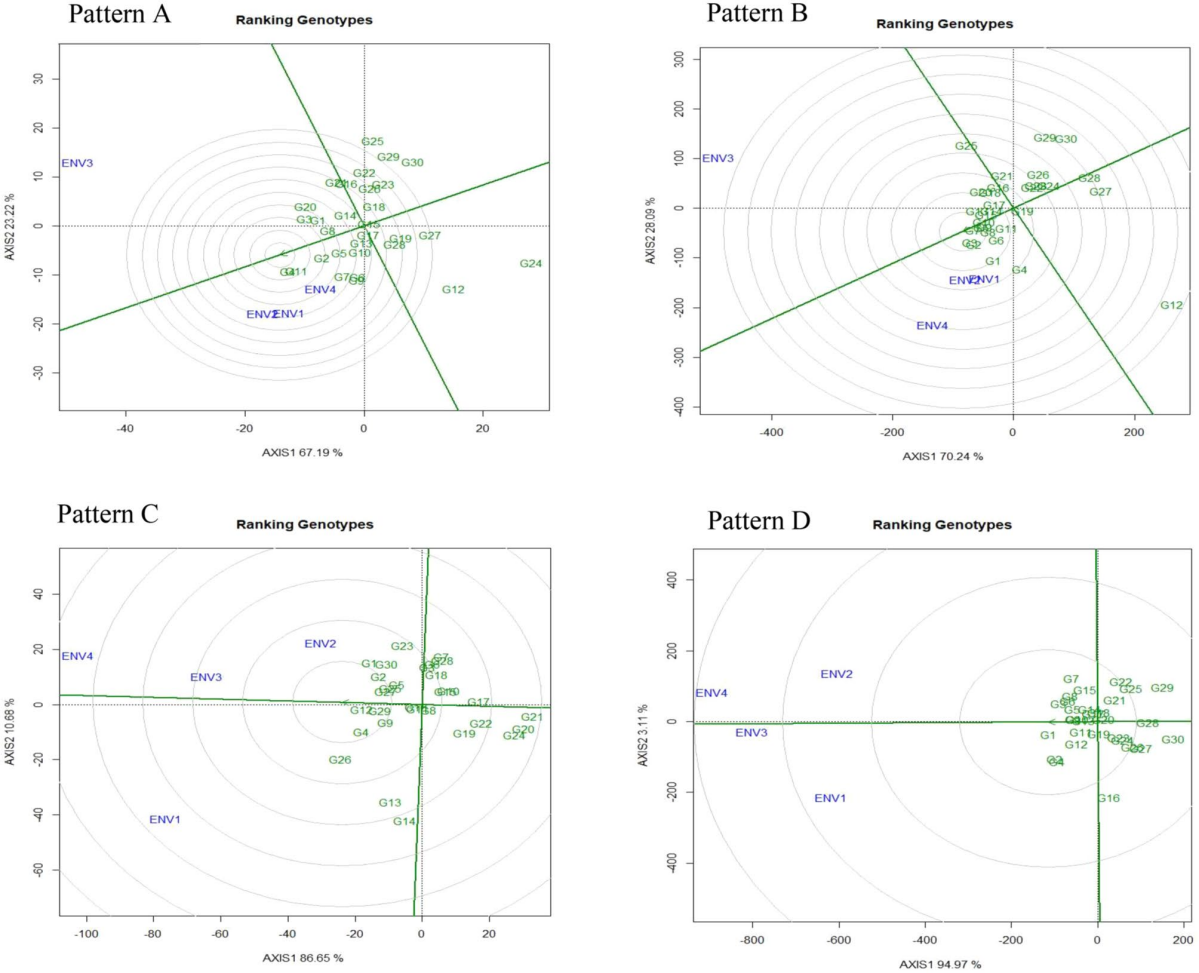
The GGE biplot ‘genotypes ranking’ pattern for genotype comparison with ideal genotype showing G + G × E interaction effect of 30 V*. subterranea* genotypes under two season two location for total number of pods (Pattern A), fresh pod weight (Pattern B), hundred seed weight (Pattern C), yield per hectare (Pattern D). The biplots were created based on Centering = 0, SVP = 2, Scaling = 0. The ideal genotype is signified by a circle within innermost concentric circles on average environment coordinate (AEC) abscissa which passed through biplot origin.

The average environment coordinate (AEC) or average environment axes (AEA) line crosses through the biplot's origin if SVP = 1 (single value portioning). As a report by Yan and Rajcan^43^, the mean of PC1 and PC2 of the environmental scores is defined. The ‘Mean vs. stability’ view frequently stating to as AEC and SVP that helps to simplify the genotype assessment based on the mean performance and stability under a wide range of environment (Fig. 2). The two straight lines, (i) the AEC abscissa (vertical) and (ii) AEC ordinate (horizontal) comprise this biplot graph. Line one (Fig. 3: Pattern A, B, C, D) consists of a single arrow that pointed towards greater mean performance for each trait. In our investigation, the ‘mean vs. stability’ pattern of GGE biplot revealed 90.41% for total number of the pod (Pattern A), 98.33% for fresh pod weight, 97.33% for hundred seed weight, and 98.08% for yield per hectare of G + G × E variation (Fig. 2). The arrow sign on the AEC abscissa line directed the ranking of genotypes in increasing order with a greater value of traits evaluated. However, genotype G4 produced higher pods followed by G11 and G2 in ENV 1, ENV 2, and ENV 4 while in ENV3, the high pod producing genotype is the G3. For the trait TNP, the genotypes G2 and G8 are more stable over the tested environment though these genotypes produced lower pods (Fig. 2; Pattern A). The highest fresh pod weight (g) was recorded for genotype G2 afterward G3 and G11 in ENV1, ENV 2, and ENV 4 but genotype G13 gave higher FPW in ENV3. Over the environment genotype G7, G10, and G15 leading to highly stable ones with lower performance Fig. 2; Pattern B). In environment one (ENV1) genotype G26 noted for HSW followed by G4 and G12 on the other hand genotype G1 showed higher HSW in ENV2, ENV 3, and ENV4 though genotype G12 and G29 considered as highly stable across the environment (Fig. 2; Pattern C). In the case of yield per hectare genotype, G1 gave higher yield followed by G2 and G4 in ENV1 and ENV3 whereas genotype G3 followed by G5, G6, G7, and G8 produced maximum yield in the ENV2 and ENV4. Among the accessions, genotype G1, G3, and G5 gave higher yield per hectare and highly stable while G10, G13, G11, G14, G17, G18, and G20 also remarked as more stable genotypes but exhibited low yielding performance (Fig. 2; Pattern D). However, these genotypes might be incorporated in the breeding strategy for crop enhancement. Aside from these, genotypes G2, G4, G6, G8, and G7 provided somewhat desired yield but shown low stability due to their position on the biplot far from the AEC line. Similar trends of observations were recorded by Oladosu et al*.*^19^, Hashim et al*.*^41^, and Sabri et al*.*^44^. However, the stability of each genotype measure by line two which crosses over the biplot origin, and it vertical bisects the AEA abscissa. The genotype positioned into nearness to the concentrical rings, determining the best performing genotype and the projection from AEA abscissa indicate the genotype stability. Genotypes consider being more stable when it placed on the horizontal axis (AEC abscissa) and had zero projection from the vertical axis (AEC ordinate) while the genotype with the lengthiest direction from the AEC abscissa is treated as unstable, a similar report was stated by Oladosu et al*.*^19^.

##### Genotype ranking: best and ideal genotype assessment

Through the genotype ranking biplot (Fig. 3) we can detect an ideal genotype in contrast to other genotypes evaluated. The genotypes G4, G11, and G2 could be noted as the best leading genotype due to their nearness to the arrowhead in the circle for total number of pods (Fig. 3: Pattern A). Similarly, for fresh pod weight genotype G2, G3, G7, G8, G9, G10, and G14 (Fig. 3: Pattern B); for hundred seed weight genotype G12, G29, and G4 (Fig. 3: Pattern C) and for yield per hectare genotype G1, G10, G13, G5, and G3 (Fig. 3: Pattern D) regarded as best genotype due to proximity to concentric circle. Commonly, an ideal genotype is always placed into the innermost circle and relatively nearer the head of the arrow at the center of the circular ring (Fig. 3: Pattern A, B, c, and D). The genotype located in the inner circle is highly desirable compared to the genotypes of the outer circle. However, in some cases no genotype was positioned inside the inner circle, consequently, genotypes next closer to the inner circle are considered to be an ideal one^19^. Consequently, genotypes G4 and G11 for TNP; genotypes G7, G9, and G10 for FPW; genotype G12 for HSW; and genotypes G1 and G10 for yield per hectare were regarded as ideal genotypes across the tested environment because they were positioned closer to the centre of the biplot origin, indicating that they are stable genotypes. For an effective selection, an ideal genotype should have both high mean and stability properties^45^. A ring at the head of the arrow on the horizontal AEC abscissa axis generally represents an ideal genotype^19^ and additionally, the idealness of a genotype refers to a small circle on the AEC abscissa line. Genotypes on the left side of the vertical line often outperform the grand mean, whereas genotypes on the right side underperform the grand mean^19^. Plant breeders used data from yield performance evaluations based on mean and stability to choose genotypes best suited to a specific environment within a multi-environment^13^, while genotypes close to the ideal genotype were also more promising or appropriate. So, the genotype ranking based on ideal genotype for yield per hectare was G1 > G10 > G13 > G5 > G3 > G6 > G14 > G17 > G11 > G12 > G8 > G2 > G4 (Fig. 3: Pattern D). Oladosu et al.^19^ found similar findings across 10 settings as evidence of our result.

#### ‘Descriminitiveness vs. representativeness’ pattern of GGE biplot

**Figure 4:**
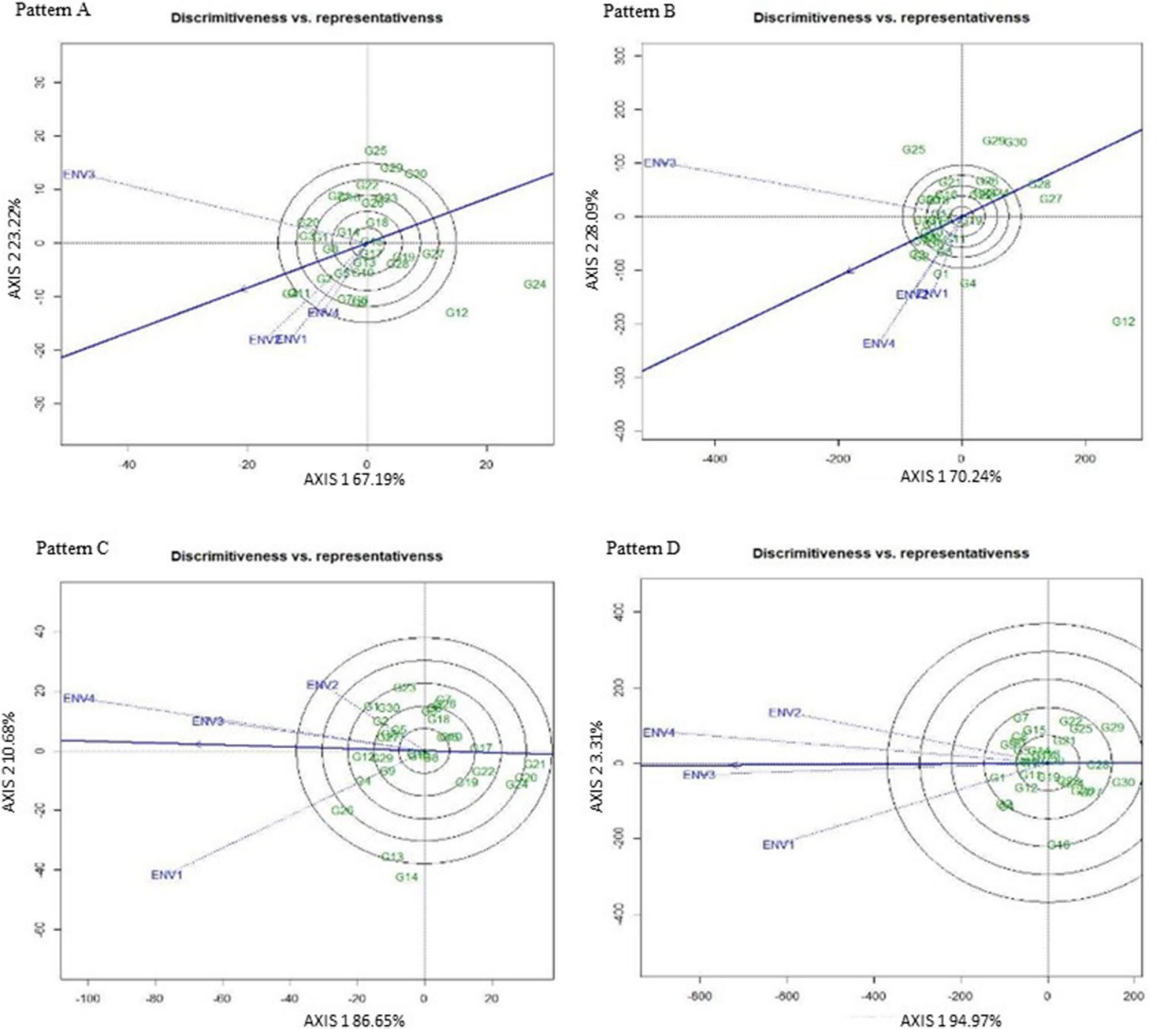
The GGE biplot ‘Descriminitiveness vs. Representativeness’ pattern for genotype comparison with ideal genotype showing G + G × E interaction effect of 30 V*. subterranea* genotypes under two season two location for total number of pods (Pattern A), fresh pod weight (Pattern B), hundred seed weight (Pattern C), yield per hectare (Pattern D). The biplots were created based on Centering = 0, SVP = 2, Scaling = 0.

The determination of a best suited (ideal) test environment is crucial for a successful breeding technique in the selection of superior genotypes. The two features like descriminitiveness (the ability of an environment to distinguish genotype) and representativeness (the ability of an environment to represent all other evaluated environments) signify the idealness of the tested environments^19^. In our investigation Fig. 4 (Pattern A, B, C, D) illustrated the ‘descriminitiveness vs. representativeness’ of the GGE biplot study.

**Figure 5:**
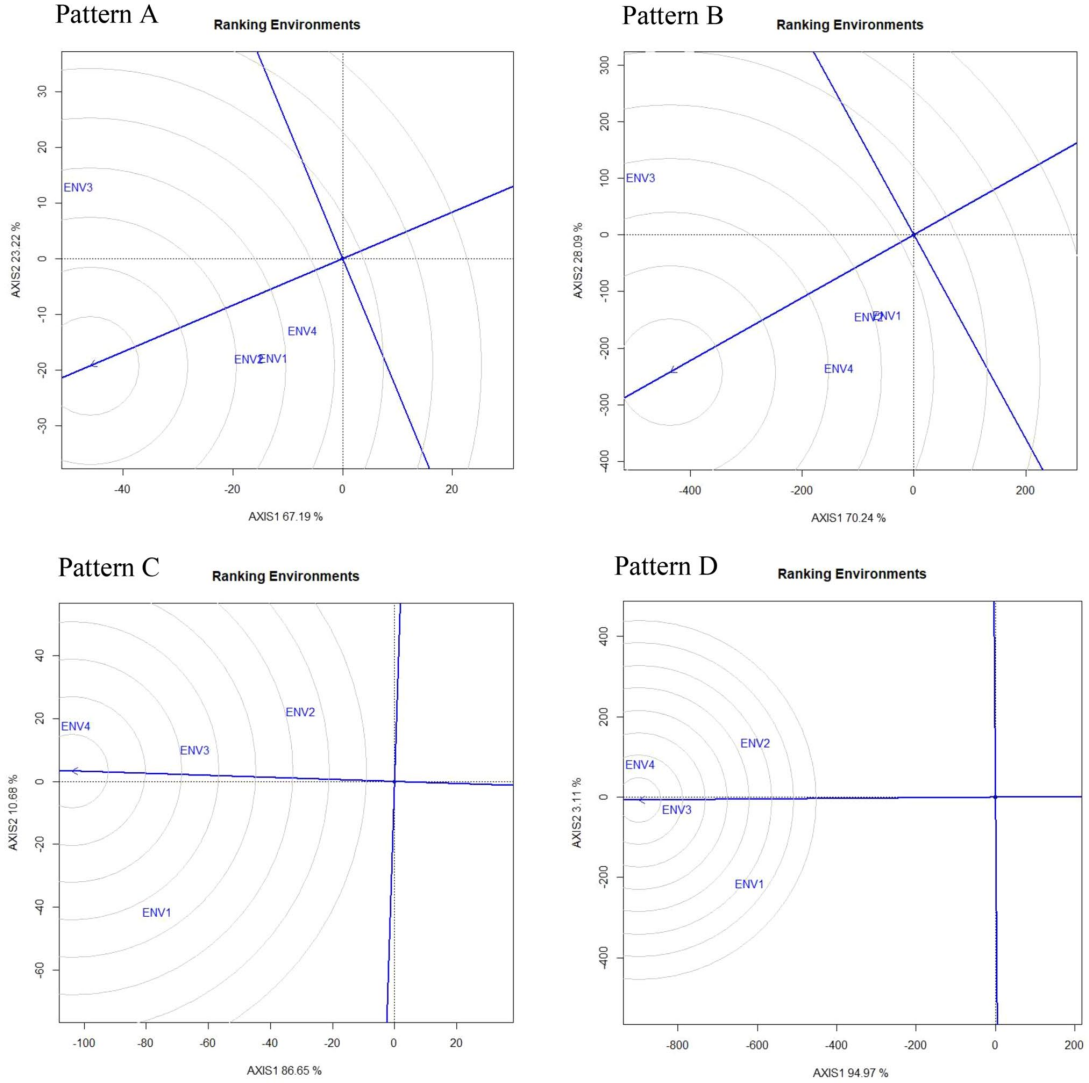
The GGE biplot ‘Environment ranking’ pattern for environment comparison with ideal environment showing G + G × E interaction effect of 30 V*. subterranea* genotypes under two season two location for total number of pods (Pattern A), fresh pod weight (Pattern B), hundred seed weight (Pattern C), yield per hectare (Pattern D). The biplots were created based on Centering = 0, SVP = 2, Scaling = 0.

[19]We recorded environment four (ENV4) for TNP (Fig. 4: Pattern A), environment (ENV1 and ENV2) for FPW (Fig. 4: Pattern B), environment two (ENV2) for HSW (Fig. 4: Pattern C), and environment (ENV1 and ENV2) for yield (Fig. 4: Pattern D) as an independent and unique research location due to their short vector while the environment with long vector is more influential in discriminating among the Bambara groundnut accessions. However, the environment with a long vector that forms a shorter angle with the AEC abscissa line is idyllic for the selection of superior genotypes. Thus, environment two (ENV2) for TNP and environment four (ENV4) for FPW, HSW, and yield had small-angle alongside long vector with AEC abscissa indicated that the test environment was greater representative and discriminative. Figure 5 represent the ranking of environment, exposed that environment ENV2 for the total number of pods (Fig. 5: Pattern A), environment ENV4 for fresh pod weight (Fig. 5: Pattern B), hundred seed weight(Fig. 5: Pattern C), and yield (Fig. 5: Pattern D) are regarded as the ideal environment. Oppositely, for TNP and FPW the environment ENV3 as well as for HSW and Yield the environment (ENV 1 and ENV 2) was noted as the poorest environment to select genotype across the environment. Thus, this study suggests that the studied genotype determined the most suitable environment to assess the mega environment based on test environments representativeness and discriminating ability. Hashim et al*.*^41^ reported one environment is ideal for genotype selection considering yield per hectare among the tested four environments. Among the five evaluated locations, three were noted as an ideal location by Oladosu et al*.*^19^. The correlation coefficient between the genotype mean value over the environment and the genotype values in that environment is approximately equal to the cosine of the angle between the average environment coordinate (AEC) often refers as the average environment axis (AEA) and the environment vector^38^. The smaller angle between AEC abscissa and vector of test environment represent the better environment related to those generate greater angles. The arrow on the AEC abscissa line shows its direction and a small concentric circle denotes the average value of the environment while the length of the test environment vector guesses the discriminating ability. The length of each environment vector gives an idea of its greatness (discriminating ability) to distinguish genotypes in the environment^19^.

#### The relatedness among the test environments: environment assessment

**Figure 6:**
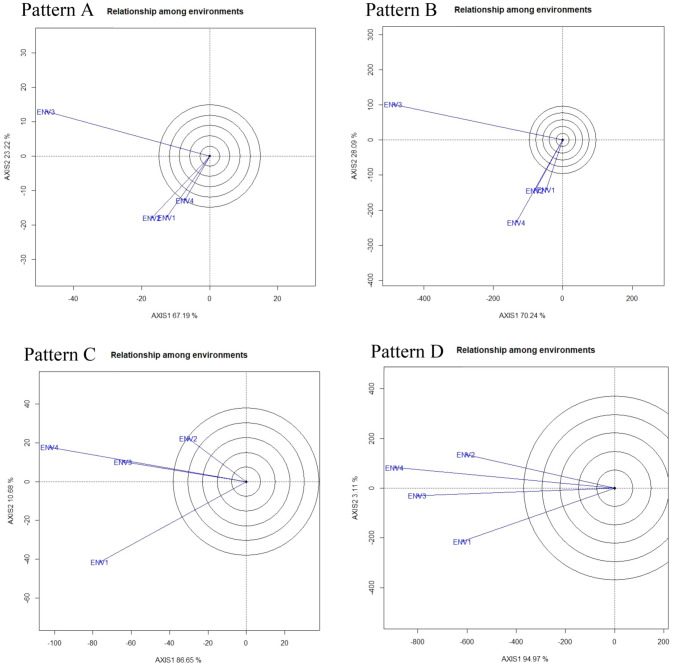
The vector view of GGE biplot showing the relationship among environment (tested environment with the ideal environment) of 30 V*. subterranea* genotypes under two season two location for total number of pods (Pattern A), fresh pod weight (Pattern B), hundred seed weight (Pattern C), yield per hectare (Pattern D). The biplots were created based on Centering = 0, SVP = 2, Scaling = 0.

Based on the biplot graph, assessment of test environment is the next important step after multi environment identification to fix the environment discriminativeness and representativeness ability, inter-relatedness, and redundant among the environments. Figure 6: Patterns (A, B, C, D) represent the discriminativeness and representativeness of tested locations. The biplot accounted for 67.19% (PC1) and 23.22% (PC2) for total number of pods, 70.24% (PC1) and 28.09% (PC2) for fresh pod weight (g), 86.65% (PC1) and 10.68% (PC2) for hundred seed weight (g) and 94.97% (PC1) and 3.11% (PC2) for yield of G + G × E interaction variation across the tested environment. In all cases, the 1st principal components showed the maximum variation for all traits evaluated. Across the location, season, and year the trait yield per hectare is largely influenced by the genotype by environment effect. The distance among each tested environment is displayed in Fig. 5 also for all evaluated traits. As a report by Lin and Binns^46^ the effect of environment on genotype is highly influenced by unpredictable (e.g., weather) and predictable (e.g., soil) factors. The soil is a fixed factor due to its persistence from year after year and is noted as a predictable component. Contrary, the weather is a complex component because it includes predictable elements that are well-defined by the overall climatic region whereas the unpredictable components arise variation due to alternation of time (year to year)^46^.

However, it is effective and productive to take into consideration evaluating test environment due to it does represent proximate to multi environments, hence, can be a representative of a multi environment^38^. Based on our findings, we categorized the tested environment into three groups. The ENV4 for TNP, ENV1, and ENV2 for FPW, ENV2 for HSW, and yield per hectare are branded as the category-1 environment (short vector) which provided little or no information on genotypes also unfit for use as a test location. Environment two (ENV 2) for TNP and FPW as well as the environment (ENV3 and ENV4) for HSW, and Yield per hectare are designated as the category-2 (lower angle vs. longer vector) indicated that these environments are appropriate for promising genotypes selection because of their notable representativeness and discriminating power. Yan and Rajacan^43^ noted the category-2 environment (long environmental vector with short angle) is a model environment even though it is close to several environments, it is effective and productive to consider assessing test environment^38^. The category-3 environment includes environment (ENV1 and ENV2) for TNP and environment (ENV2 and ENV4) for FPW whereas environment (ENV1) for HSW and Yield per hectare should not be appropriate for superior genotype selection whatever it is suitable for detecting unstable genotypes. Yan et al.^38^ categorized the environment into three principal categories based on discriminativeness and representativeness during his study. Hashim et al*.*^41^ reported one environment is suitable for genotype selection considering yield per hectare among the tested four environments.

### Additive main effects and multiplicative interaction: AMMI 1

**Figure 7:**
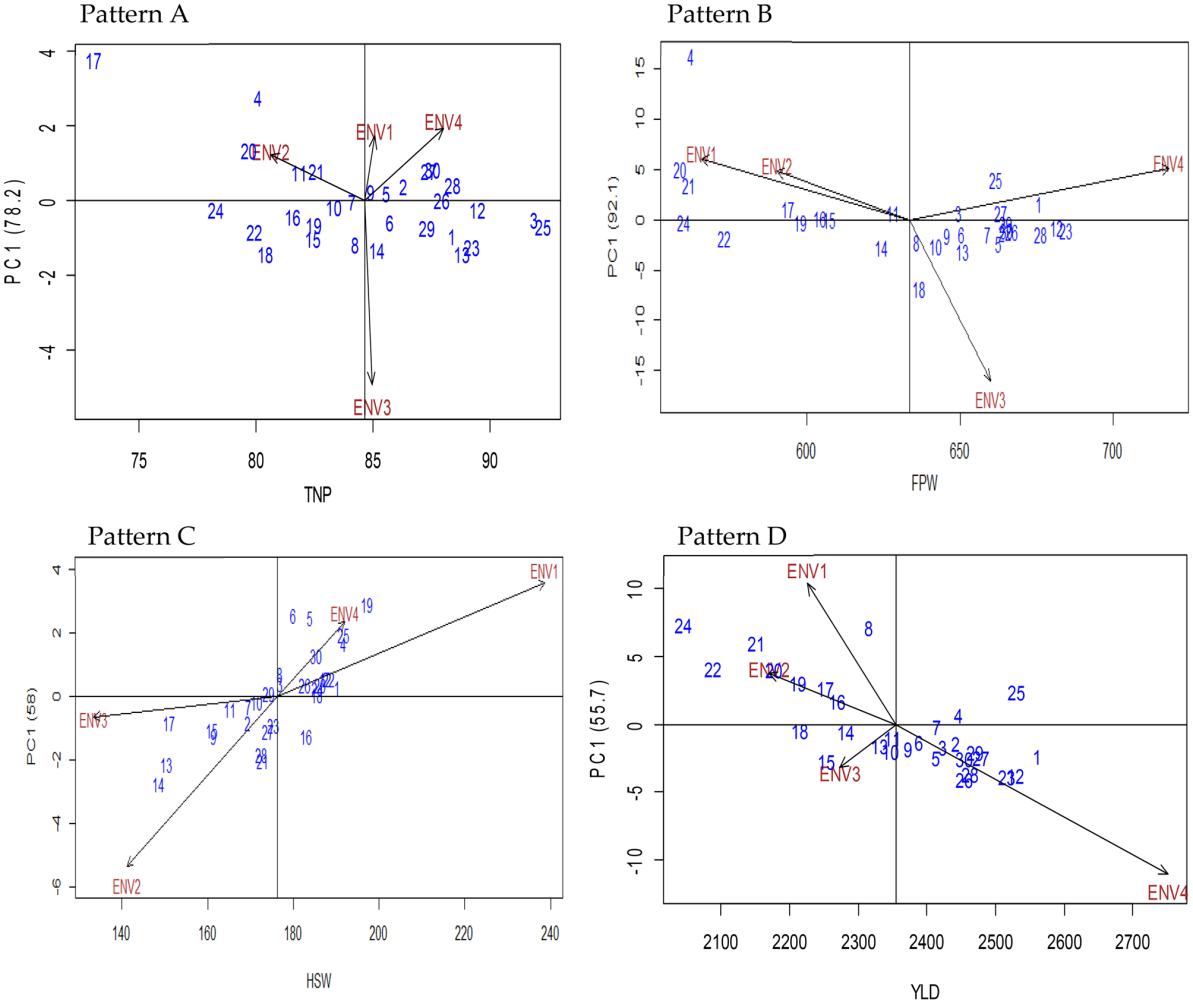
The biplot ‘AMMI 1’ illustrated the trait main effect and first principal component (PC1) effects of both genotype and environment of 30 V*. subterranea* genotypes under two season two location for total number of pods (Pattern A), fresh pod weight (Pattern B), hundred seed weight (Pattern C), yield per hectare (Pattern D). The biplots were created based on Centering = 0, SVP = 2, Scaling = 0.

In additive main effects and multiplicative interaction 1 (AMMI 1), the biplot abscissa and ordinate indicated the 1st principal component (PC1) term and the trait's significant influence, respectively. In this study, Fig. 7 (Pattern A, B, C, and D) showing the additive main effects and multiplicative interaction 30 genotype and 4 environments for the trait TNP, FPW, HSW, and yield per hectare, respectively. Based on genotype mean and interaction with the environment a little similarity was found among the genotypes. For hundred seed weight (Fig. 7: Pattern C) environment three (ENV3), ENV2 for total number of pods (Fig. 7: Pattern A), ENV4 for fresh pod weight (Fig. 7: Pattern B), and ENV2 for yield per hectare (Fig. 7: Pattern D) had a PCA1 score or vector closer to zero compared to other environments, indicates lower interaction effect which almost ensures the better performance of all genotypes in that environment. Moreover, these environments are treated as suitable for all genotypes evaluated. For total number of pods the genotypes G24, G10, G7, G9, G5, G26, and G12 (Fig. 7: Pattern A); for fresh pod weight the genotypes G24, G19, G10, G15, G17, G11, G3, G27, and G29 (Fig. 7: Pattern B); for hundred seed weight the genotypes G10, G29, G18, G21, G1(Fig. 7: Pattern C); for yield per hectare the genotypes G18, G14, G7, G3, G1, G5, and G4 (Fig. 7: Pattern D) had approximately zero scores on the first PCA1 axis which indicates that these genotypes were less influenced by the environment. However, some genotypes had their mean below-average performance though, in general, plant breeders are highly attracted to genotypes that are high-yielding and relatively more stable. Genotypes with PC1 scores adjacent to zero lines of biplot indicated that genotypes were suited to all environments, whereas PC1 vectors with the same sign and score but away from zero lines of biplot indicated that genotypes were adapted to a specific environment, is supported by Murphy et al.^47^. When the PCA1 score for a genotype or environment is near to zero, there is a small interaction impact; contrary, if a genotype and environment achieve the same sign on the PCA axis, there is a positive interaction; otherwise, there is a negative interaction. A report published by Mogale^11^ is comparable to our findings in Bambara groundnut and Oladosu et al.^19^ in rice.

#### Additive main effects and multiplicative interaction: AMMI 2

**Figure 8:**
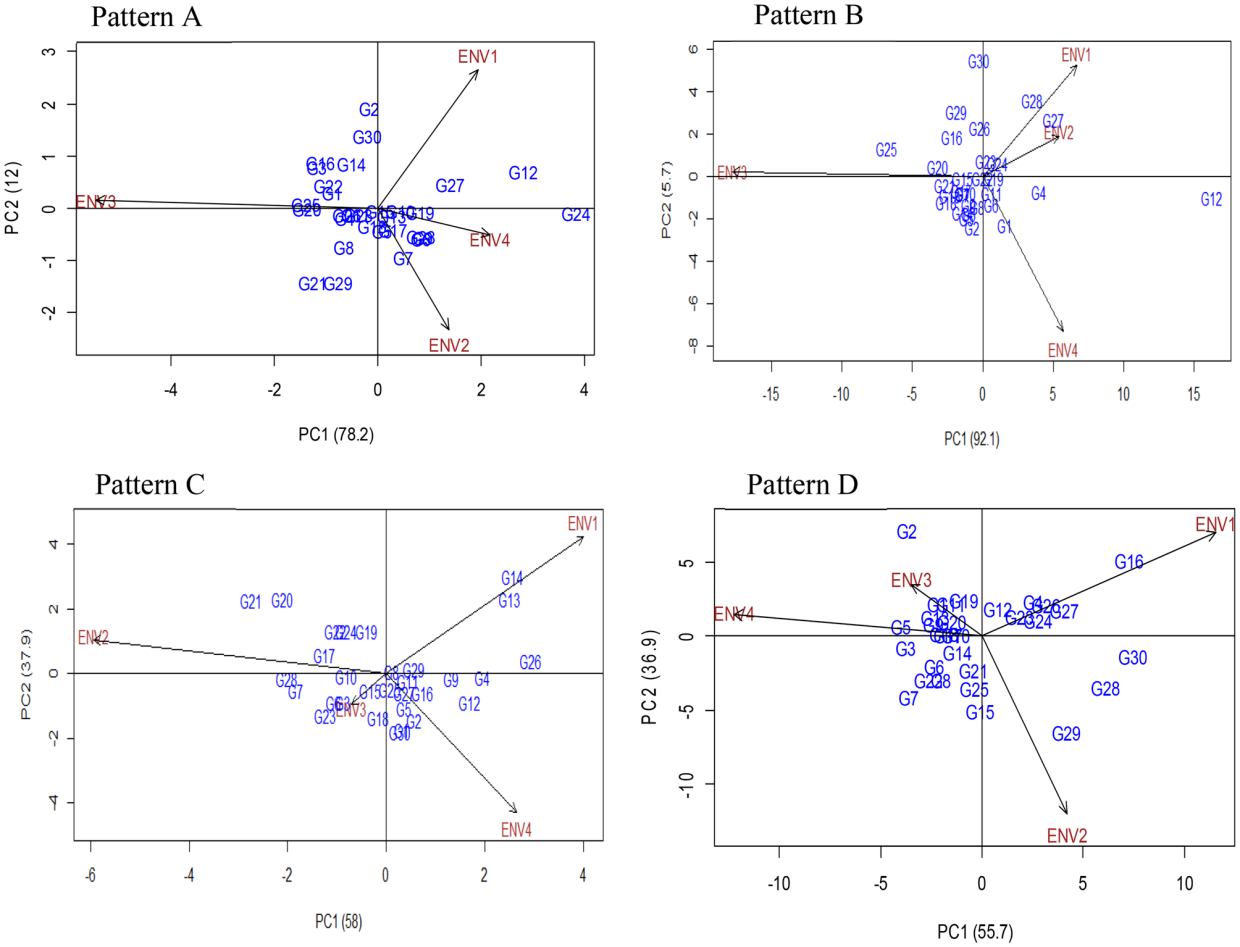
The biplot ‘AMMI 2’ illustrated the first two principal components (PC1 and PC2) effects of genotype plus GE interaction effect of 30 V*. subterranea* genotypes under two season two location for total number of pods (Pattern A), fresh pod weight (Pattern B), hundred seed weight (Pattern C), yield kg/ha (Pattern D). The biplots were created based on Centering = 0, SVP = 2, Scaling = 0.

Additive main effects and multiplicative interaction 2 (AMMI 2) is a principal component (PC1 and PC2) scores-based graphical representation of summarized information which gives advantages over joint regression-based analysis. The AMMI 2 divulge and inferring the complicated GEI that involves significant multi-environments and detection of genotypes with either broad or narrow spectrum adaptability. Figure 8 illustrated the first two principal component interaction of the AMMI 2 biplot model which accounted for 90.02%, 97.8%, 95.9%, and 92.6% of the G + G × E interaction variation for the total number of pods (Fig. 8: Pattern A), fresh pod weight (Fig. 8: Pattern B), hundred seed weight (Fig. 8: Pattern C) and Yield per hectare (Fig. 8: Pattern D), respectively. Accordingly, this proportion of variation implies that interaction of 30 V*. subterranea* genotypes that tested in four environments was projected by 1st two principal components of genotype and environment. Our result was consistent with the endorsement of Gauch and Zobel^42^ stated that the first two PCs are sufficient for the projection of the AMMI model precisely oppositely, some researchers namely, Sivapalan et al.^48^ and Tariku et al.^49^ suggested 1st four PCs to report the multi-environment trail. The center of the biplot (0, 0) is divided into four distinct sectors by passing through the two-line, vertically, and horizontally (Fig. 8). Likewise, GGE biplot, genotypes that are placed apart from the biplot origin is regarded as winning genotypes in the environments that fall in that sector. The extent of interaction revealed by the environment over the genotype and vice versa is controlled by the distance of the environment and genotype vectors that originated from the origin of the biplot. In our study, we found ENV4 for TNP (Fig. 8: Pattern A), ENV2 for FPW (Fig. 8: Pattern B), ENV3 for HSW (Fig. 8: Pattern C), and yield per hectare (Fig. 8: Pattern D) had short vector comparatively other environments. As a report by Murphy et al*.*^47^ the environment indicator with a shorter vector i.e., nearer to biplot origin is less interactive and treated as a perfect index for selecting genotype with mean performance and adaptability. Most of the genotypes for the traits TNP, FPW, HSW, and Yield per hectare were assembled (Fig. 8: Pattern A, B, C, D) close to the biplot origin. However, genotypes that assembled or cluster together on the biplot origin indicating that genotypes have identical feedback to all tested environments compared to the genotypes that are positioned away from each other, this statement is corroborated with the report of Akter et al.^50^. Moreover, genotypes that are placed apart for biplot origin are more sensitive to environmental interaction related to closely positioned genotypes to biplot origin. The correlation coefficient and the degree of interaction of genotype and environment can be highlighted by the angle between the vectors of environment and genotype. There was no correlation when environment and genotype form a right-angle while the severe and nearer angle between them indicates negative and positive correlation, respectively. The findings in this current investigation have authorized statements reported by Oladosu et al*.*^19^ using two seasons five locations.

### Evaluation of genotypes

In this study, the evaluation of the selected genotype for yield and its positively correlated contributing trait’s stability over a wide range of environments are justified through the result of a significant test of GE interaction.

#### Mean performance and comparison of genotypes

The means comparison and average performance of 30 V*. subterranea* genotypes over four environments were listed in Table 5. Over the location, all the genotypes showed significant variation for total number of pods (TNP), fresh pod weight (FWP), hundred seed weight (HSW), and Yield per hectare. The total number of pods ranged from 73 (G24) to 92 (G4) with an average of 8484.67 ± 0.46. The genotype G4 produced the highest number of pods (92) followed by G11 (91), G2, and G3 (89) though across the genotype it was 106 while the lowest was 51. The highest fresh pod weight was 684.58 g for genotype G3, followed by G2 (681.56 g), and the lowest was 58.78 g for the genotype G27. The maximum fresh pod weight over the four environment was 810.22 g, while the minimum was 289.64 g, with an average of 633.61 g. The greater value of hundred seed weight was accounted for 197.16 g (G5) followed by 191.69 g (G2) though the lower value was 148.45 g for G28. The average weight of hundred seeds was 176.32 ± 2.43 with a range of 97.97 g to 339.43 g over tested environments. In terms of yield per hectare, genotype G1 had the highest yield (2560.29 kg/ha), followed by genotypes G4 (2530.05 kg/ha) and G2 (2528.33 kg/ha), while genotype G30 had the lowest yield of 045.12 kg/ha. However, yield per hectare varied from 1854 to 3160 kg/ha, with an average of 2354.59 kg/ha throughout the studied environments.

**Table 5:** Mean performance and their comparisons of *V. subterranean* genotypes for yield and yield contributing traits over four environments (two seasons at two locations).

Genotype	TNP	FPW	HSW	YLD
G1	88.38a,c	675.71a–c	190.31a–c	2560.29a
G2	89.46a,b	681.56a,b	191.69a,b	2528.33a,b
G3	89.24a,b	684.58a	182.91b–i	2515.5a–c
G4	92.26a	661.73c–g	185.44b–g	2530.05a,b
G5	87.93a–d	666.89b–d	197.16a	2454.53c–e
G6	87.41a–e	663.27c–g	186.30a–e	2477.99b–d
G7	88.39a–c	676.35a–c	172.79h–l	2463.37b–d
G8	87.32a–e	664.91c–f	188.2a–d	2469.92b–d
G9	87.56a–d	665.29c–e	185.57b–f	2454.7c–e
G10	86.28b–f	663.74c–g	187.52a–d	2441.21d–f
G11	91.88a	649.59g–j	175.34e–k	2422.21d–f
G12	80.09h–j	562.16n	191.75a,b	2445.45c–e
G13	85.56b–h	662.51c–g	182.66b–i	2412.93d–g
G14	85.73b–g	650.49f–j	174.02g–k	2388.67e–h
G15	84.12b–i	658.95d–h	172.50h–l	2413.4d–g
G16	84.21b–i	635.88j–l	174.28f–k	2315.42i–l
G17	84.8b–i	645.95h–j	185.39b–g	2372.86f–i
G18	83.31c–j	642.01i–k	169.43j–m	2347.23g–j
G19	81.84e–j	628.15k,l	176.95d–j	2348.94g–j
G20	88.81a–c	650.71e–i	183.94b–h	2330.09h–k
G21	85.15b–i	624.272l	180.07c–j	2281.46j–m
G22	82.41d–j	607.48m	169.64j–m	2253.82l,m
G23	81.58f–j	604.18m	176.9d–j	2269.25k–m
G24	73.05k	593.95m	161.51l–n	2251.74l,m
G25	80.39g–j	636.66i–l	171.47i–m	2214.62m,n
G26	82.47d–j	597.98m	165.13k–m	2211.42m,n
G27	79.70i,j	558.78n	150.42n,o	2177.63n
G28	82.60d–j	561.53n	148.45o	2151.62n,o
G29	79.92i,j	573.23n	160.97m,n	2087.88o,p
G30	78.31j,k	559.89n	150.98n,o	2045.12p
Mean ± SE	84.67 ± 0.46	633.61 ± 4.44	176.32 ± 2.43	2354.59 ± 14.86
LSD	5.57	14.68	11.51	72.01
CV	10.41	13.32	26.24	11.98
Std. Dev	8.82	84.41	46.26	282.13
Max. (across environment)	106.50	810.22	339.43	3160.00
Min. (across environment)	51.90	289.64	97.97	1854.00

*TNP* total number of pods, *FPW* fresh pod weight (g), *HSW* hundred seed weight (g), *YLD* yield (kg/ha), *CV* coefficient of variation, *Se*. standard error, *Std. Dev.* standard deviation, *Max.* maximum, *Min.* minimum.  Means within each column with the same letter are not significantly different with LSD test at p > 0.05.

For plant breeders, yield and its contributing attributes such as TNP, FPW, and HSW may be important agronomic factors for identifying superior cultivars in Bambara groundnut^11^. Most of the time, these traits have a strong positive association with yield. According to the findings of Khan et al.^3^ and Khan et al.^4^, the traits viz. TNP, FPW, and HSW are primary component characteristics that have a dominant influence on yield due to their positive significant relationship with yield. Lowering the number of pods resulted in decreased fresh pod weight, as well as fewer dried pod weight and seeds, all of which badly influence grain yield in Bambara groundnut^3,11^. A genotype that is stable to yield in a diversified environment, on the other hand, is highly accepted by any researchers in a breeding programme to reduce the danger of yield loss owing to adverse climatic conditions^19^. In such a situation, when genotype performance is inconsistent in a diverse environment, the study of GEI followed by stability analysis is crucial which is advocated by Haldane^51^ and Baye et al*.*^52^.

## Conclusion

The main intention of this current multi-environmental study is to evaluate V. subterranea genotypes based on mean performance under a wide range of environments in order to identify superior genotypes. The multi environmental trail (MET) of Bambara groundnut genotypes may also give information on genotype adaptability and stability to a certain environmental situation. Eventually, a genotype is proposed for commercial cultivation, its susceptibility to genotype by environment interaction (GEI) should be assessed. However, considering the multivariate (GGE and AMMI biplot) statistical result the tested genotypes are categorized into three major groups. Group one genotypes are those that are highly stable and have a high yielding potential. This group comprises genotypes of G1, G3, and G5, which are well suited to a range of environments. The basic criteria for the second category are genotypes with low stability but high yield per hectare. This group contains genotypes of G2 and G4 (perform better in ENV1), as well as G6, G8, and G7 (perform better in ENV2) that are appropriate for a specific environment. The last group worked with genotypes that had a low yield but a high stability. This group comprises genotypes of G10, G13, G11, G14, G17, G19, and G18, which are ideal for breeding schemes intended to improve certain phenotypes. This category of genotypes may have yield component compensation criteria, such as the ability to recover quickly from a wide range of environmental challenges. Genotypes G1, G3, and G5 performed well across all test locations and designated as ideal in terms of mean, stability, high yield, and emerged as the top genotype among those investigated. Grain yield and its contributing characteristics (total number of pods, fresh pod weight, hundred seed weight, and so on) are strongly influenced, either directly or indirectly, by a variety of environmental factors. Our findings suggested that breeding may improve bambara groundnut production efficiency, and that ideally-established genotypes could be recommended for commercial cultivation in Malaysia as well as in tropical region.
