# Author Correction: AMMI and GGE biplot analysis for yield performance and stability assessment of selected Bambara groundnut (*Vigna subterranea* L. Verdc.) genotypes under the multi-environmental trials (METs)

**DOI:** 10.1038/s41598-022-11781-w

**Published:** 2022-05-03

**Authors:** Md Mahmudul Hasan Khan, Mohd Y. Rafii, Shairul Izan Ramlee, Mashitah Jusoh, Md Al Mamun

**Affiliations:** 1 Laboratory of Climate-Smart Food Crop Production, Institute of Tropical Agriculture and Food Security (ITAFoS), Universiti Putra Malaysia (UPM), 43400 Serdang, Selangor Malaysia; 2 Department of Crop Science, Faculty of Agriculture, Universiti Putra Malaysia (UPM), 43400 Serdang, Selangor Malaysia; 3 Bangladesh Agricultural Research Institute (BARI), Gazipur, 1701 Bangladesh; 4 Bangladesh Jute Research Institute (BJRI), Dhaka, Bangladesh

Correction to: *Scientific Reports* 10.1038/s41598-021-01411-2, published online 23 November 2021

The original version of this Article contained an error in the title of the paper, where the word “trials” was incorrectly given as “trails”. The original Article has been corrected.

